# Comprehensive Geriatric Assessment as a predictor model for post-operative delirium in older adults undergoing major non-cardiac elective surgery

**DOI:** 10.3389/fmed.2025.1473459

**Published:** 2025-07-17

**Authors:** Anastasia Asylia Dinakrisma, Kuntjoro Harimurti, Rudyanto Sedono, Ikhwan Rinaldi, Nina Kemala Sari, Pradana Soewondo, Hamzah Shatri, Sukamto Koesnoe

**Affiliations:** ^1^Division of Geriatrics, Department of Internal Medicine, Cipto Mangunkusumo National General Hospital - Faculty of Medicine Universitas Indonesia, Jakarta, Indonesia; ^2^Department of Anesthesiology, Cipto Mangunkusumo National General Hospital - Faculty of Medicine Universitas Indonesia, Jakarta, Indonesia; ^3^Department of Internal Medicine, Cipto Mangunkusumo National General Hospital - Faculty of Medicine Universitas Indonesia, Jakarta, Indonesia

**Keywords:** comprehensive geriatric assessment, post - operative delirium, elective major surgery, older adults, prediction model

## Abstract

**Background:**

The needs for surgery in older adult populations is increasing every year. Postoperative delirium is one the most common complications and will impact many adverse outcomes. Comprehensive Geriatric Assessment (CGA) and perioperative risk stratification of older adults are needed as an initial prevention strategy as well as an efficient and applicable prognosis predictor model.

**Objectives:**

This study aims to determine the incidence of post-operative delirium and develop a prediction model for delirium in older adults after major non-cardiac elective surgery based on predictor factors.

**Methods:**

This research is a retrospective cohort study using secondary data from medical records of older adult inpatients who underwent major elective non-cardiac surgery at Cipto Mangunkusumo Hospital between January 2020 and March 2023. Data analysis using SPSS 20.0 and STATA 10. Development of a prediction model for post-operative delirium complications using the Hosmer- Lemeshow test and Area Under the Curve of the Receiver Operating Characteristic (AUC ROC).

**Results:**

Total of 370 subjects that met the criteria were analyzed. The incidence of post-operative delirium was 6.8%. The predictor factors analyzed were age (HR = 3.43; 95% CI 1.544–7.635), cognitive status (HR = 2.74; 95% CI 1.156–6.492), and nutritional status (HR = 3.35; 95% CI 1.459–7,679). The postoperative delirium complication prediction model had good calibration (*p* > 0.05) and moderate score performance for predicting the incidence of delirium in older adults [AUC 0.750 (*p* < 0.001; 95% CI 0.640–0.860)].

**Conclusion:**

Age, cognitive status, and nutritional status are strong predictors of postoperative delirium in older adults undergoing major non-cardiac elective surgery. The postoperative delirium prediction model has good calibration and moderate score performance.

## Background

It is estimated that more than half of all operations are performed at the age of 65 years or older. Studies in England from 1999 to 2015 recorded an increase in the number of surgical procedures from 14.9% to 22.9% in the population aged over 75 years (1–3).

Post-operative neurocognitive disorder is a cognitive impairment that occurs more than 30 days or is still diagnosed up to 12 months after surgery. The incidence of cognitive impairment post non-cardiac surgery is 26% and decreases by 10% within 3 months post-surgery. Post-operative delirium is usually assessed within 24 h to 5–7 days after surgery, and it can be either persistent or recurrent delirium (4). Post-operative delirium has an impact on many poor outcomes, such as prolonged length of stay and treatment costs, increased postoperative morbidity, high rates of rehospitalization within 30 days, decreased functional status, decreased quality of life, and short-term and long-term mortality which was observed to reach 4.5 years after major surgery (5–7).

Several studies show that 30%–40% of postoperative delirium events can be prevented (8). The initial strategy in preventing delirium is to identify various predisposing factors, possibly perioperative precipitating factors through risk stratification assessments, so that appropriate preventive measures, anticipatory steps, and management of post-operative delirium can be taken (9). To date, several instruments are available to calculate the risk stratification of postoperative delirium by including Comprehensive Geriatric Assessment (CGA) components, such as functional status, nutritional status, cognitive impairment, and polypharmacy with other perioperative risk factors components, such as comorbidities, type of surgery, electrolyte disturbances, albumin, blood glucose, serum urea creatinine, and ASA score (10–14). Nevertheless, perioperative risk classification of older persons that evaluates variables from CGA, such as depression and frailty status, as well as intraoperative and post-operative variables as predictors of prognostic models, has shown inconsistent results in the previous 10 years.

Another important thing that needs to be considered in a risk stratification instrument, apart from its good calibration and discrimination capabilities, is its ability to be applied in various clinical settings. Risk stratification of post- operative delirium in older adults that has been used at Cipto mangunkusumo National Hospital is Marcantonio’s score. However, this score cannot be implemented in its entirety, because it uses a fairly complex assessment of cognitive status with the TICS (Telephone Interview for Cognitive Status) examination which includes 11 questions, and a history of alcohol abuse, which is less relevant to conditions in Indonesia. Basic Health Research Data in Indonesia in 2018 shows that the number of older adult people with alcohol abuse is only 0.5% of the population in Indonesia (10, 15). Murdianis et al. (16), conducted the same study, but did not include intra- and post- operative factor. The CGA assessment used was complicated so it was less applicable.

Therefore, the development of an efficient and applicable risk stratification assessment for postoperative delirium in older adults is still very necessary. This study aims to assess various components of CGA more completely, such as comorbidities, functional status, cognitive status, depression status, nutritional status, polypharmacy, frailty status, and other perioperative factors which include pre-, intra-, and post-operative factors, as risk factors for post-operative delirium.

## Materials and methods

This study is a retrospective cohort study taken from medical records of inpatients who underwent major elective non-cardiac surgery at Cipto Mangunkusumo National General Hospital. This research has passed ethical review from the Health Research Ethics Committee, Faculty of Medicine, Universitas Indonesia, with registered No. KET- 559/UN2.F1/ETIK/PPM.00.02/2023. The sample used in this study was 370 older adults who underwent major elective non-cardiac surgery in the period January 2020–June 2023. Consecutive searches of medical records of patients who met the inclusion criteria were carried out as a sampling method from the reachable population. The probability sampling method is carried out by using random sampling on the study population. Patient’s data were analyzed by two independent researchers to minimize biases.

Inclusion criteria were patients aged equal to or above 60 years who underwent major non-thoracic-cardiovascular elective surgery. Major non-thoracic-cardiovascular elective surgery were classified based on Modified-John Hopkins Surgical Criteria grade II and III (17), without cardio- thoracic surgery. Delirium pre- and post- operative was assessed based on subjective data from medical record (keywords: “confusion,” “agitation,” “disorientation,” “delusional”) and/ or objective data with CAM (Confusion Assessment Method) score conducted by health care professionals (7, 18). Functional status was assessed with Activity Daily living Barthel Index, cognitive status with MiniCOG or Abbreviated Mental Test, depression status with Geriatric Depression Scale five items or 15 items, nutritional status with Mini Nutritional Assessment Short Form or Full Form, frailty status with F.R.A.I.L score or Clinical Frailty Scale version 2.0. Polypharmacy described as the regular use of five or more medications (19). Post-operative delirium defined as delirium that occurs 24 h–7 days after surgery (7, 18).

Exclusion criteria are incomplete data, if the CGA domain data is incomplete, patients with incomplete intraoperative and postoperative data, and patients with a history of delirium before surgery.

Data were analyzed by using SPSS 20.0 and STATA 10. Cox proportional hazards model was performed; independent variables with *p*-values < 0.25 in the bivariate Cox regression test will be included in the multivariate Cox regression test. If the independent variable meets the PH assumption, a multivariate test with Cox regression will be used. A prediction model for post-operative delirium complications was developed with the Hosmer- Lemeshow test and Area Under the Curve of the Receiver Operating Characteristic (AUC ROC).

## Results

This study analyzed 370 data from patients who underwent major elective non-cardiac surgery from January 2020 to June 2023. The characteristics of research respondents can be seen in Tables 1–3 below.

**TABLE 1:** Characteristics of research subjects.

Variable	Total (*N* = 370)
**Gender, n (%)**	
Man	144 (39.5)
Woman	224 (60.5)
Age (year). median (IQR)	66 (63–69
Waiting time for the day of surgery (day). median (IQR)	17 (5–38)
Surgery duration (minute). median (IQR)	210 (135–290)
Sedation duration (minute), median (IQR)	270 (185–360)
**Caregiver status, n (%)**	
Yes	25 (6.8)
No	345 (93.2)
**Surgery Type, n (%)**	
Orthopedic surgery	94 (24.4)
Neurosurgery	24 (6.5)
Oncology	40 (10.8)
Gynecology	59 (15.9)
Urology	21 (5.7)
Digestive	59 (15.9)
ENT	38 (10.3)
Vascular	18 (4.9)
Others	17 (4.6)
**In hospital death, n (%)**	
Yes	195 (52.7)
No	175 (47.3)
Comorbidities, *n* (%)	
Diabetes mellitus	96 (25.9)
Hypertension	169 (45.7)
Cardiovascular disease	48 (13)
Chronic kidney disease	42 (11.4)
Cerebrovascular disease	12 (3.2)
Malignancy	136 (36.8)
Multimorbidity (≥ 2 diseases)	258 (69.7)
**Anesthesia type, n (%)**	
General anesthesia	209 (56.6)
Regional anesthesia	128 (34.6)
Mixed	31 (8.4)
**ICU/HCU post operation, n (%)**	
Yes	160 (43.2)
No	210 (56.8)
**In hospital death, n (%)**	
Yes	2 (0,5)
No	368 (99,5)
**Indication of pre-rehabilitation, n (%)**	
Yes	192 (5.9)
No	178 (8.1)
**Pre-rehabilitation is carried out, n (%)**	
Yes	93 (48.4)
No	99 (51.6)

**TABLE 2:** Characteristic of research subject based on Comprehensive Geriatric Assessment (CGA) variables.

Variable	Total (*N* = 370)
**Age, n (%)**	
≥ 70 years	92 (24.9)
≤ 70 years	278 (75.1)
**Functional status, n (%)**	
Moderate – total dependency	40 (10.8)
Independent – mild dependency	330 (89.2)
**Cognitive status, n (%)**	
Probability of cognitive disturbance	38 (10.3)
No disturbance	332 (89.7)
**Depression status, n (%)**	
Probability of depression	60 (16.2)
No depression	310 (83.8)
**Nutritional status, n (%)**	
Risk of malnutrition – malnutrition	114 (30.8)
Normal	256 (69.2)
**Frailty status, n (%)**	
Frail	69 (18.6)
Fit-prefrail	301 (81.4)
**Polypharmacy or use of anticholinergic effect or benzodiazepine drugs, n (%)**	
Yes	79 (21.4)
No	291 (78.6)
**ASA status, n (%)**	
≥ 3	136 (36.8)
0–2	234 (63.2)
**Type of surgery, n (%)**	
Orthopedic	94 (25.4)
Non-orthopedic	276 (74.6)
**Electrolyte imbalance (sodium, potassium, glucose), n (%)**	
Yes	61 (16.5)
No	309 (83.5)
**Intraoperative hypotension, n (%)**	
Yes	87 (23.5)
No	283 (76.5)
**Perioperative pain (Visual Analog Score/VAS score), n (%)**	
≥ 4	84 (22.7)
0–3	286 (77.3)
**Post-operative hematocrit, n (%)**	
< 30%	92 (24.9)
> 30%	278 (75.1)

**TABLE 3:** Characteristics of research subjects with post-operative delirium complications.

Variable	Total (*N* = 25)
Onset. median (day) (RIK)	1 (1–4)
Delirium duration (hour). median (RIK)	96 (15–216)
**Delirium type, n (%)**	
Hypoactive	7 (28)
Hyperactive	9 (36)
Mixed	9 (36)
**Healthcare who diagnose delirium, n (%)**	
Medical Doctor	8 (32)
Nurse	2 (8)
Both	15 (100)
**Medication used to treat delirium, n (%)**	
Haloperidol	7 (28)
Others	1 (4)
No drug	17 (68)
**Restrain**	
Yes	5 (20)
No	20 (80)
**Medical instruments [catheter, nasogastric tube (NGT)], n (%)**	
Yes	23 (92)
No	2 (8)
**HCU/ICU admission post-surgery**	
Yes	24 (96)
No	1 (4)

Multicollinearity analysis was carried out by correlating the independent variables one by one. Based on the multicollinearity test and correlation test, none of the determinants of 30 days post-surgical complications met the collinearity criteria, so further analysis could be carried out. The proportional hazard (PH) assumption is calculated to assess whether the comparison of the rate of occurrence of an event between groups at any time is the same. The independent variables included in the subsequent analysis have proportional hazard assumptions that are met (global test > 0.05). If the Global test is not met, but the Kaplan Meier test and the Ln [-Ln (survival)] test, are met, the PH assumption is considered not met. The results of the Proportional Hazard assumption analysis results show that all independent variables meet the PH assumption so that bivariate and multivariate analysis can use Cox regression. Table 4 shows cox bivariate regression analysis of independent variables and post-operative delirium.

**TABLE 4:** Cox bivariate regression analysis of independent variables and post-operative delirium.

Variable	Delirium		HR (95% IK)	*P*-value
	**Yes**	**No**		
**Age, n (%)**				
≥ 70 years	14 (15.22)	78 (84.78)	3.901 (1.771–8.595)	*0.01
< 70 years	11 (4.00)	267 (96.00)	–	–
**Functional status, n (%)**				
Moderate – total dependency	7 (17.50)	33 (82.50)	3.082 (1.286–7.386)	*0.012
Independent – mild independency	18 (5.45)	312 (94.55)	–	–
**Cognitive status, n (%)**				
Probability of cognitive disturbance	8 (21.05)	30 (78.95)	4.169 (1.797–9.669)	*0.001
No disturbance	17 (5.12)	315 (94.88)	–	–
**Depression status, *n* (%)**	**6 (10.0)**	**54 (90.0)**	**1.639 (0.655–4.106)**	**0.291**
Probability of depression	19 (6.13)	291 (93.87)	–	–
No depression	–	–	–	–
**Nutritional status, n (%)**				
Risk of malnutrition - malnutrition	16 (14.04)	98 (85.96)	3.899 (1.722–8.831)	*0.001
Normal	9 (3.52)	247 (96.48)	–	–
**Frailty status, n (%)**				
Frail	8 (12.50)	56 (87.50)	2.263 (0.967–5.292)	*0.059
Fit-prefrail	16 (5.32)	285 (94.68)	–	–
**Polypharmacy, n (%)**				
Yes	6 (7.59)	73 (92.41)	1.168 (0.466–2.925)	0.740
No	19 (6.53)	272 (93.47)	–	–
**ASA status, n (%)**				
≥ 3	12 (8.82)	124 (91.18)	1.553 (0.708–3.403)	0.272
0–2	13 (5.56)	221 (94.44)	–	–
**Surgery type, n (%)**				
Orthopedic	7 (7.45)	87 (92.55)	1.127 (0.480–2.699)	0.788
Non-orthopedic	18 (6.52)	258 (93.48)	–	–
**Sodium, potassium, and glucose disorder n (%)**				
Yes	5 (8.20)	56 (91.80)	1.282 (0.481–3.417)	0.619
No	20 (6.47)	289 (95.35)	–	–
**Intraoperative hypotension, n (%)**				
Yes	10 (11.49)	77 (88.51)	2.040 (0.915–4.547)	*0.081
No	15 (5.30)	268 (94.70)	–	–
**Post-operative pain, n (%)**				
≥ 4	6 (7.14)	78 (92.86)	0.972 (0.387–2.441)	0.953
0–3	19 (6.64)	267 (93.36)	–	–
**Hematocrit post-operation, n (%)**				
< 30%	8 (8.70)	84 (91.30)	1.375 (0.593–3.186)	0.458
> 30%	17 (6.12)	261 (3.88)	–	–

*p < 0.25 were included in multivariate analysis.

Variables that have *p* < 0.250 in the bivariate analysis are continued in the initial multivariate Cox Regression model until *p* < 0.05 is obtained in the final multivariate model or which are considered clinically important will be selected as independent predictors of the incidence of post- operative delirium. The six variables included in the multilevel multivariate Cox regression were age, functional status, cognitive status, nutritional status, frailty status, and intraoperative hypotension.

Multivariate analysis is needed to determine independent predictor factors. In multivariate analysis with the Cox Proportional Hazard Regression Model, variables with *p* < 0.05 and clinically important variables were obtained. Table 5 is a stepwise multivariate survival analysis using backward LR methodology which includes six variables in the initial model until variables are obtained that meet the *p*-value < 0.05, namely age, cognitive status, and nutritional status.

**TABLE 5:** Multivariate analysis of independent variables for delirium post-operative complications.

Variable	HR (95% CI)	*P*-value
**First step**	–	–
Age	3.435 (1.489–7.923)	0.004
Functional status	0.765 (0.240–2.436)	0.650
Cognitive status	2.482 (0.902–6.828)	0.078
Nutrition status	2.982 (1.187–7.491)	0.020
Frailty status	1.215 (0.476–3.101)	0.684
Intraoperative hypotension	1.878 (0.812–4.346)	0.141
**Second step**		
Age	3.491 (1.518–8.031)	0.003
Functional status	0.805 (0.258–2.515)	0.710
Cognitive status	2.503 (0.907–6.906)	0.076
Nutrition status	3.087 (1.249–7.491)	0.003
Frailty status	1.892 (0.819–4.370)	0.136
**Third step**		
Age	3.471 (1.552–7.762)	0.002
Functional status	2.474 (1.018–6.009)	0.045
Cognitive status	3.146 (1.357–7.294)	0.008
Nutrition status	1.718 (0.753–3.919)	0.198
**Last model**		
Age	3.434 (1.544–7.635)	0.002
Cognitive status	2.740 (1.156–6.492)	0.022
Nutrition status	3.347 (1.459–7.679)	0.004

The creation of a scoring system for post-surgical complication factors was carried out using calculations. Hazard Ratio (HR) and rounded to the nearest value. Table 6 displays the steps for creating a scoring system.

**TABLE 6:** Establishing delirium factor scoring system.

Variable	HR	Score rounding
**Age** ≥70 years	3.434	3
**Cognitive status** (mini COG 0–2, or AMT < 8)	2.740	3
**Nutritional status** (MNA SF 0–11)	3.347	3

Based on this score, age, cognitive status, and nutritional status each have a score weight of three. The total score ranged from 0 to 9. Table 7 displays the probability of 30 days post-operative complications for each total score.

**TABLE 7:** Probability of post-operative delirium for each score.

Total score	Delirium		Total	Probability
	**Yes**	**No**		
0	5	176	181	1.84
3	7	135	142	6.61
6	8	31	39	21.16
9	5	3	8	50.43

Based on the sensitivity and specificity values, a cut point value of six can be obtained so that the probability of a patient experiencing post-operative delirium can be calculated. The quality and performance prediction score that has been created is assessed through calibration (using the Hosmer-Lemeshow test) and its discrimination ability by looking at the area under the receiver operating characteristic curve (AUC) value.

In the score system calibration test using the Hosmer-Lemeshow test, p was obtained at 0.369 where the score system had good calibration based on the statistical significance of the Hosmer-Lemeshow Test (*p* > 0.05).

**FIGURE 1:**
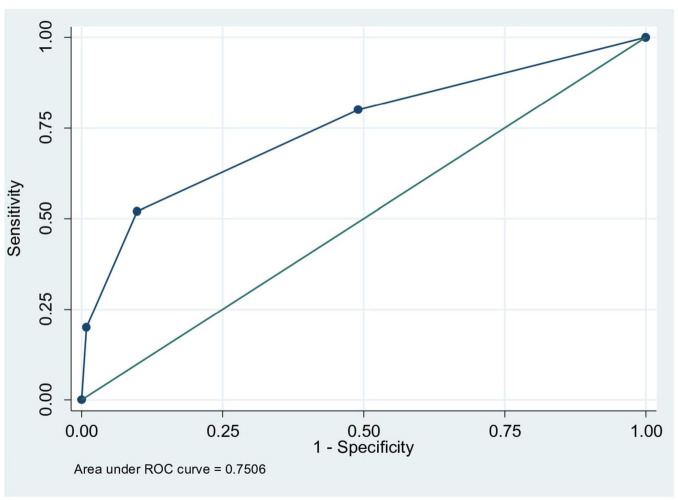
Area under the curve (AUC) of variable prediction model for post-operative delirium.

Testing the score system on the ROC (Receiving Operator Characteristics) curve shown in Figure 1 obtained an AUC of 0.750 (*p* < 0.0001, 95% CI 0.640–0.86), showing the score performance results which is used to predict the incidence of delirium in older adults.

## Discussion

In January 2020–March 2023, there were 2,243 undergoing surgeries, with 60.1% undergoing major surgery at Cipto Mangunkusumo Hospital, Jakarta. This shows that major surgical procedures in the older adult are a need that cannot be avoided and are likely to increase in the future (3, 12).

In this study, age over 70 years was a predictor of postoperative delirium with an adjusted HR of 3.43 (95% CI 1.54–7.64). These results are in line with studies from Vochteloo et al. (13), Wang et al. (14), showing that age over 70 years increases the risk of post-operative delirium with OR 1.03, 95% (CI 0.99–1.07) and OR 1.090 (95% CI 1.021–1.163 *p* = 0.009). Chronological age serves as a surrogate variable for the accumulation of various risk factors associated with aging, which may increase the risk of post-operative delirium. The declined of organ functions, accompanied with increased sensitivity to anesthetic drugs (especially general anesthesia), contains of GABA receptor antagonists, play a role in disturbance of circadian rhythms (sleep-wake cycles) (15).

The findings of this study showed that in bivariate analysis, impaired functional status increased the risk of delirium with crude HR 3 (95% CI 1.2–7.38), but it was not significant in multivariate analysis. These findings are different from the study conducted by Litaker et al. (17) which showed a decrease in functional status causing post-operative delirium, while the study by Robinson et al. (18) showed the same result, but was not a strong predictor factor compared to with cognitive impairment, comorbid factors and hematocrit on multivariate analysis. Impaired functional status describes a person’s comorbid burden and physiological potential. Study by Gabriel et al. (19) showed that simple operation can reduce a person’s intrinsic capacity by 40% so individuals with decreased preoperative functional capacity will experience a greater burden on surgical stressors.

Cognitive status in this study was assessed using miniCOG or AMT which was a strong predictor of post-operative delirium with an adjusted HR of 2.7 (95% CI 1.15–6.4). The study by Galanakis et al. (20), showed that cognitive impairment and a previous history of dementia increased the risk of post-operative delirium, cognitive impairment with an MMSE score < 24 increased the risk of postoperative delirium by 1.32 times, and the study from Robinson et al. (18), with a mini COG score < 3 was a strong predictor of postoperative delirium with an RR of 2.8 (< 0.001). The process of surgery and anesthesia can trigger an inflammatory response in the central nervous system which is triggered by inflammatory processes in the periphery (activation of macrophages, neutrophils and various pro-inflammatory cytokines). This metabolic imbalance process can cause various disorders in the hippocampus system, microglia, astrocytes and cerebral blood vessels, in the form of neuroinflammatory processes, decreased perfusion and micro embolism which results in decreased cognitive function, known as post-operative cognitive dysfunction (POC) (21).

Depression status in this study was not a predictor of postoperative delirium. This finding contrast with the study by Lenze et al. (22), which showed depression increased the risk of postoperative delirium. Depression triggers the body’s stress response which will increase the response of inflammatory mediators in the brain, circadian rhythm disturbances which are associated with the pathophysiology of post-operative delirium (23). The insignificance of depression in this study is probably because the incidence of delirium is not much different from the group without depression which has other stronger predictor factors.

The risk of malnutrition and malnutrition in this study are strong predictors of post-operative delirium with an adjusted HR of 3.347 (95% CI 1.45–7.67). These results are in line with the study by Mazzola et al. (24), where the risk of experiencing delirium was 2.9 times higher for those with malnutrition, and even reached 6.3 Nutritional status will likely affect the patient’s tolerance for stress due to surgery, post-operative physical recovery, wound healing and post-surgical infections. Nutritional disorders will affect the humoral and cellular immune systems. Cellular immunity is impaired by decreasing the number of T cells in lymphoid tissue, decreasing the chemotactic response of neutrophils, cleaning bacteria, bactericidal function of neutrophils, inhibiting the response of inflammatory cells to the site of infection, including decreasing complement activity. Immunity disorders are related to deficiencies of various trace elements such as zinc, pyridoxine, vitamin A, B12, C and folate in conditions of malnutrition (25).

In this study, frailty was not a strong predictor of post-operative delirium. This finding is different from the study by Mahanna-Gabrielli et al. (26), which showed that prefrail and frail phenotypes assessed with the FRAIL score examination increased the odds of postoperative delirium by 2.7 times and the systematic review study by Gracie et al. (27), showed a pooled OR of 2.1. The results of this study are comparable to the results of research by Murdianis et al. (16), which showed that the results were not significant. It is possible that there was a small incidence of delirium in this study.

Polypharmacy, the use of anticholinergic effects drugs, and benzodiazepine class drugs are not predictors of post-operative delirium. This is different from several studies, such as the Goldenberg et al. (28), which showed that the use of more than three types of drugs increases the risk of post-operative delirium by three times (OR 3.6, 95% CI 1.9–5.9), a study by Hermann et al. (29) in 2022 showed that the use of anticholinergic drugs, as indicated by the anticholinergic burden score, increases the risk of post-operative delirium by 2.7 times (OR = 2.74, 95% CI = 1.55–4.94), and the study by Murakawa et al. (30), in 2015, showed that the use of pre-operative benzodiazepine drugs increased the risk of delirium by 3.9 times (odds ratio, 3.97; 95% CI, 1.09–14.5; *p* = 0.03). This may be due to the small number of delirium incidents and the small combined number of polypharmacy, anticholinergic, and benzodiazepine users, making the incidence of delirium insignificant between the two groups.

Elevated inflammatory biomarkers are considered to be a cause of post-operative complications in frail patients. In surgical conditions there is an increase in levels of acute phase inflammatory proteins, such as CRP and Interleukin-6, an increase in oxidative stress (reactive oxygen species), an increase in free radicals which induce transcription of inflammatory mediators and dysregulation of the immune system. These things will cause further decline in organ function in frailty patients and increasingly severe cases of homeostasis dysregulation (31). The prefrail and frail phenotypes are associated with post-surgical complications including delirium, increased length of stay, increased risk of morbidity, increased rehospitalization, and mortality within 30 and 90 days (31, 32).

This study shows that ASA status is not a risk factor for postoperative delirium. This finding is different from study by Bilge et al. (33), which showed that an ASA score in older adults was associated with a risk of delirium of 3.3 times and the study by Raats et al. (34), which showed an ASA score three or more increased the risk of post-operative delirium by 2.6. This due to difference in this study, ASA score was no more than three. The ASA (American Society of Anesthesiologists) score aims to assess pre-anesthesia conditions associated with comorbidities. This score together with other conditions such as type of surgery, frailty, functional status can be useful for predicting perioperative risk.

The type of surgery in this study was not a risk factor for post-operative delirium. In contrast to research from Albanese et al. (35) with a risk of delirium in pelvic surgery of 4%–53%, Mcalpine et al. (36) with a risk of delirium in gynecological surgery of 17.5%, Xu Y et al. (15) with a risk of delirium in intracranial surgery of 12%–26%, Brown et al. (37) with a risk of delirium in cardiovascular thoracic surgery of 26%–54%. In this study, the incidence of delirium in orthopedic surgery was 7%, while in non-orthopedic surgery it was 6%. This difference may be due to the study including all types of surgery except cardiovascular surgery. Type of surgery is associated with increased inflammatory biomarkers associated with the pathophysiology of post-operative delirium. Meanwhile, the results of this study show that sodium, potassium and glucose disorders are not a risk factor for post-operative delirium. In contrast to the study by Marcantonio et al. (10) in 1994, which showed that sodium levels < 130 or > 150 mmol/L, potassium < 3 or > 6 mmol/ L, and blood glucose < 60 or > 300 mg/ dl were associated with a risk of delirium of 2.8 times. Preoperative electrolyte and glucose disturbances likely reflect systemic decompensation of the patient’s medical condition and are associated with a slow recovery phase. In this study, it is possible that the disorder can still be compensated systemically as indicated by an ASA score of three or less.

The findings of this study show that intraoperative hypotension is not a strong predictor of postoperative delirium, although it is still included in the multivariate analysis with *p* < 0.25. These are different from research conducted by Rahim et al. (38) which showed that systolic blood pressure < 90 mmHg for a duration of 1 min or more was associated with a 1.6 times increase in the risk of delirium, a duration of ≥ 5 min increases the risk by five times, and the duration hypotension ≥ 10 min increases the risk to nine times. This may be due to other stronger predictor factors in the final model in this study such as nutritional status, age and cognitive status, whereas Rahim et al. (38) research included different variables as confounders.

This study showed that hematocrit and postoperative pain were not risk factors for postoperative delirium. A meta-analysis study from Smith et al. (39) showed that intra- and post-operative factors such as post-operative hematocrit and post-operative pain did not significantly influence the incidence of post-operative delirium (*p* ≥ 0.12) but the studies were heterogeneous.

The incidence of post-operative delirium in this study was 6.8%. Table 3 showed the most common types of delirium in this study were hyperactive delirium (36%) and mixed delirium (36%), followed by hypoactive delirium (7%). The median onset of delirium was post-operative day 1–day 4, with a median duration of delirium of 96 h (15–216 h). Several studies have shown that hypoactive and mixed types of delirium carry a worse prognosis in terms of mortality during treatment. On subjective examination, hypoactive delirium tends to be under-diagnosed due to its quiet clinical manifestations. Hypoactive delirium is associated with older adult who are frailer and more dependent so they are clinically more vulnerable, caregivers have difficulty recognizing the problem, and lower tolerance for rehabilitation or physical activity (40). A study from Pisani et al. (41) in ICU patients with delirium showed that the duration of delirium was associated with an increased risk of mortality within 1 year.

The scoring system was tested using the ROC curve. The result is that the area under the curve (AUC) has a value of 0.75, *p* < 0.0001, CI 95% (0.640–0.86) indicating moderate performance of the score for predicting the incidence of delirium in older adults. The *Hosmer Lameshow discriminiation* test revealed good discrimination, with p 0,369 (*p* > 0.05). The scoring system are a combination of three components, which are age, nutritional status and cognitive status. The Marcantonio scoring system has an AUC value of 0.81 ± 0.04, but consist of more components, such as age > 70 years, alcohol use, cognitive assessment with Telephone Interview for Cognitive Status (TICS) score, functional status, abnormal sodium, potassium, and glucose levels, and the type of non-cardiac or aortic aneurysm surgery (10). The scoring system of this study is simpler than Marcantonio score, with moderate performance and good calibration. In patients with moderate to high probability of postoperative delirium, preventive intervention for optimizing preoperative preparation, intraoperative management, and postoperative care should be done by an interdisciplinary team. The selected internal validation method is the bootstrapping technique. The bootstrapping results are consistent with the previous analysis results, so that the internal validation of this research is good.

The limitation of this study is that it has a retrospective design based on data in medical records. It may cause biases due to incomplete recorded data and limit the control of some variables and the ability to establish causality. Thus, it is necessary to carry out further research with a prospective design in order to better control the variables studied, with a larger number of samples with multi-center studies. The result of this research, can be applied in the setting of older populations who will undergo major non-cardiac surgery in tertiary hospital. Generalization of research results in other populations and clinical settings requires further validity testing.

## Conclusion

The proportion of older adults who experienced delirium complications after major non-cardiac elective surgery at Cipto Mangunkusumo Hospital is 6.8%. Age, cognitive status and nutritional status are strong predictors of post-operative delirium. A prediction model for delirium after major non-cardiac elective surgery was obtained which included components of age, cognitive status and nutritional status, with a moderate score performance for predicting the incidence of post-operative delirium, as well as good calibration and internal validation.

## Data Availability

The raw data supporting the conclusions of this article will be made available by the authors, without undue reservation.
